# Health-related quality of life in subjective cognitive decline and mild cognitive impairment: a longitudinal cohort analysis

**DOI:** 10.1186/s13195-023-01344-0

**Published:** 2023-11-15

**Authors:** Sandar Aye, Vincent Bouteloup, Ashley Tate, Anders Wimo, Ron Handels, Delphine Jean, Bengt Winblad, Linus Jönsson

**Affiliations:** 1 Division of Neurogeriatrics, Department of Neurobiology, Care Sciences and Society, Karolinska Institutet, BioClinicum J9:20, Akademiska Stråket, Solna, 171 64 Sweden; 2 Centre Inserm U1219, Institut de Santé Publiqued’Epidémiologie Et de Développement (ISPED), Bordeaux School of Public Health, Université de Bordeaux, 146 Rue Léo Saignat, Bordeaux, Cedex 33076 France; 3 CHU de Bordeaux, Pôle de Santé Publique, Bordeaux, 33000 France; 4 Deperatment of Psychiatry and Neuropsychology, Maastricht University, Alzheimer Centre Limburg, School for Mental Health and Neurosciences, Maastricht, the Netherlands; 5 Theme Inflammation and Aging, Karolinska University Hospital, Huddinge, Sweden

**Keywords:** Quality of life, Health utility, Subjective cognitive decline, Mild cognitive impairment, Alzheimer’s disease

## Abstract

**Background:**

Health-related quality of life (HR-QoL) is an important outcome for patients and crucial for demonstrating the value of new treatments. Health utility estimates in subjective cognitive decline (SCD) and mild cognitive impairment (MCI) are limited, especially in biomarker-confirmed populations. Besides, little is known about the longitudinal HR-QoL trajectory. This study aims to provide health utility estimates for SCD and MCI and investigate the QoL trajectory along the disease continuum.

**Methods:**

Longitudinal data from 919 SCD and 1336 MCI patients from the MEMENTO cohort were included. SCD was defined as clinical dementia rating (CDR) = 0, and MCI as CDR = 0.5. HR-QoL was measured using the EQ-5D-3L patient-reported instrument. Linear mixed-effect models (LMM) were used to assess the longitudinal change in HR-QoL and identify predictors of these changes.

**Results:**

Baseline health utilities were 0.84 ± 0.16 and 0.81 ± 0.18, and visual analogue scale (VAS) were 75.8 ± 14.82 and 70.26 ± 15.77 in SCD and MCI. In amyloid-confirmed cases, health utilities were 0.85 ± 0.14 and 0.86 ± 0.12 in amyloid-negative and amyloid-positive SCD, and 0.83 ± 0.17 and 0.84 ± 0.16 in amyloid-negative and amyloid-positive MCI. LMM revealed an annual decline in health utility of − 0.015 (SE = 0.006) and − 0.09 (SE = 0.04) in moderate and severe dementia (*P* < 0.05). There was a negative association between clinical stage and VAS where individuals with MCI, mild, moderate, and severe dementia were on average 1.695 (SE = 0.274), 4.401 (SE = 0.676), 4.999 (SE = 0.8), and 15.386 (SE = 3.142) VAS points lower than individuals with SCD (*P* < 0.001). Older age, female sex, higher body mass index, diabetes, cardiovascular history, depression, and functional impairment were associated with poor HR-QoL. Amyloid positivity was associated with an annual decline of − 0.011 (SE = 0.004, *P* < 0.05) health utility over time.

**Conclusions:**

Health utility estimates from this study can be used in economic evaluations of interventions targeting SCD and MCI. Health utility declines over time in moderate and severe dementia, and VAS declines with advancing clinical stages. Amyloid-positive patients show a faster decline in health utility indicating the importance of considering biomarker status in HR-QoL assessments. Future research is needed to confirm the longitudinal relationship between amyloid status and HR-QoL and to examine the level at which depression and IADL contribute to HR-QoL decline in AD.

**Supplementary Information:**

The online version contains supplementary material available at 10.1186/s13195-023-01344-0.

## Introduction

Alzheimer’s disease (AD) is the most common cause of dementia, accounting for 60–70% of all dementia cases [1]. The clinical spectrum of AD can be divided into cognitively unimpaired (CU) characterised by normal cognition with or without subjective cognitive decline (SCD) [2], mild cognitive impairment (MCI) without significant functional limitations [3], and dementia characterised by cognitive and functional impairment [4]. The progressive nature of the disease not only leads to increasing restrictions on the ability to perform activities of daily living (ADLs) but also affects the health-related quality of life (HR-QoL) of patients and caregivers and imposes a burden on society through care dependency [5].

The recent advancement in disease-modifying treatments (DMTs) [6] and biomarkers [7, 8] has provided the hope that people with AD will soon have access to early diagnosis and treatment that can alter the disease trajectory and maintain quality of life (QoL). While the final goal of these interventions was to improve patients’ HR-QoL by maintaining cognitive and functional abilities [9], understanding HR-QoL in SCD and MCI is essential from the patient and caregiver’s perspective. Moreover, evidence on value for money is crucial for implementing these interventions in the clinical setting and for reimbursement decisions. Therefore, understanding the QoL in SCD and MCI is important not only from the patient perspective but also from the economic perspective for the accurate evaluation of cost-effectiveness of these interventions.

HR-QoL is a multidimensional construct that can be measured by generic or disease-specific instruments [10]. These instruments can further be categorised into preference-based or non-preference-based measures. Commonly used generic preference-based instruments include EurolQol-5D (EQ-5D) [11], Health Utility Index [12], and Short form-6D [13], while the disease-specific preference-based instrument includes DEMQOL [14]. In the context of economic evaluation, generic preference-based instruments are preferred as they generate health utility values ranging from 0 (utility of being dead) to 1 (utility in perfect health), allowing for comparison across different diseases [15]. The health utility can be used to generate quality-adjusted life year (QALY) which is the important outcome measure for cost-utility analysis (CUA) [15].

Although many studies have estimated health utilities in AD, they are limited to clinically diagnosed dementia [16]. Moreover, most of the studies relied on caregivers as a proxy to rate patient’s QoL and consistently showed higher health utility ratings by patients than by caregivers [17]. A recent systematic review of health utility in the full spectrum of AD revealed limited information on health utility estimates in MCI and a lack of knowledge in SCD [17]. We identified two studies investigating health utility in SCD and MCI populations with known amyloid status. The first is the Swedish BioFINDER study, which estimated a utility of 0.87 in amyloid-positive and amyloid-negative SCD and health utilities of 0.81 and 0.71 in amyloid-positive and amyloid-negative MCI [18]. The second study evaluated the longitudinal trajectory of QoL [19], revealing a faster decline of health utilities in amyloid-positive SCD and MCI compared to their amyloid-negative counterparts, but did not provide health utility estimates. The factors influencing such decline still need to be explored.

Existing studies have reported that advanced age, female sex, poor cognition, depression, institutionalisation, and functional dependence significantly predict poor HR-QoL in individuals with dementia [20, 21]. However, institutionalisation and functional dependence are consequences of cognitive decline and are more prevalent in dementia than in the predementia stage. It remains unclear whether these predictors are equally relevant in the predementia stage. The primary aim of this study was to provide health utility estimates for SCD and MCI. The secondary aim was to investigate the longitudinal change in HR-QoL and identify the predictors of such changes.

## Methods

### Study participants

This was a longitudinal study of SCD and MCI patients enrolled in the MEMENTO cohort. The MEMENTO is a large clinic-based cohort of participants consulting in French memory clinics between April 2011 and June 2014 and presenting with either isolated cognitive complaints or recently diagnosed MCI [22]. The inclusion and exclusion criteria and the definitions for SCD and MCI used in this cohort have been described elsewhere [22]. Participants were followed at 6–12 monthly intervals for 5 years. Clinical dementia rating (CDR) [23], mini-mental state examination (MMSE) [24], Neuropsychiatric Inventory-Clinician (NPI-C) [25], Instrumental Activities in Daily Living Scale (IADL) [26], and HR-QoL [11] information were collected at baseline and each follow-up visit. Cerebrospinal fluid (CSF) examination and positron emission tomography (PET) scans are optional in this cohort. All examinations followed standardised procedures.

In this analysis, SCD was defined as CDR = 0 and MCI as CDR = 0.5. Diagnosis of dementia was based on the Diagnostic and Statistical Manual of Mental Disorders 4th edition (DSM-IV) criteria for dementia [27] and the National Institute of Neurological and Communicative Disorders and Stroke/Alzheimer’s Disease and Related Disorders Association Criteria for AD [28]. All dementia cases were reviewed by an expert panel blinded to amyloid status. Patients diagnosed with dementia were sub-divided into mild (MMSE 21–30), moderate (MMSE 10–20), and severe (MMSE < 10) dementia based on the MMSE value. Among 2323 patients enrolled in the MEMENTO cohort, 2255 patients with at least one recorded EQ-5D utility or visual analogue scale (VAS) were included in the study.

#### HR-QoL

HR-QoL was assessed using EQ-5D 3-level (EQ-5D-3L) patient-reported version [11]. It measures the participant’s health in five dimensions (mobility, self-care, usual activity, pain/discomfort, anxiety/depression) and three levels (no problem, moderate problems, and extreme problems). The responses are converted into a utility value using the France tariff [29]. The utility value ranges from 0 to 1, where 0 represents death and 1 represents perfect health. A negative utility value indicates a health state worse than death. The EQ-VAS rates the health state on a vertical visual analogue scale (VAS) from 0 to 100, where 0 represents ‘Worst imaginable health state’ and 100 represents ‘Best imaginable health state’.

#### Amyloid status

Amyloid-PET scan and CSF amyloid beta (Aβ42) were used to define amyloid status. Patients were defined as amyloid-positive if they had a pathologic amyloid-PET scan or CSF Aβ42 ELISA < 750 pg/ml, whichever comes first.

#### IADL

Lawton’s scale was used to report the impairment in IADL [26]. The scale measures function in eight domains: using telephone, shopping, food preparation, housekeeping, laundry, transportation, handling medications, and finances. Each domain was assigned between 1 point if performed independently and 4 points if unable to perform the activity. The domain scores were summed up into a single total score. Using the total IADL score will underestimate the functional impairment since domains like food preparation, housekeeping and laundry are usually not applicable in male patients leading to low IADL scores. To account for this limitation, we corrected the IADL score as suggested by Dufournet et al. [30]. The resulting IADL is an average score over the responded domains and ranges from 1 to 4, with higher scores indicating poor function and dependence.

#### Other variables

Depression was measured by the depression domain of NPI-C. The score ranges from 0 to 21, with a higher score indicating more symptoms [25, 31]. Diabetes was defined if having self-reported diabetes or anti-diabetic drug intake or glycaemia > 7 mmol/L, hypertension was defined if taking an anti-hypertensive drug or if the mean of three blood pressure measurements was either ≥ 140 mmHg for systolic blood pressure or ≥ 90 mmHg for diastolic blood pressure, dyslipidaemia was defined if taking a lipid-lowering drug or plasma cholesterol > 6.24 mmol/L, and history of cardiovascular disease was defined if having a self-reported history of myocardial infarction, surgical bypass, stroke, peripheral artery disease, or angina pectoris.

### Statistical analysis

The baseline characteristics of SCD and MCI patients were summarised in mean and standard deviation for continuous variables and frequency and percentage for categorical variables. Independent *t*-tests and chi-squared tests were used to analyse the group differences. First, we used linear mixed effect models (LMM) with random intercept to assess longitudinal change in HR-QoL. The models included EQ-5D utility and VAS as dependent variables (in separate models) and clinical stage as the independent variable. We introduced clinical stage as a time-dependent variable and used time since enrollment to denote the disease duration assuming QoL change with disease progression and over time. The interaction term of time and clinical stage was introduced in the health utility model but not in the VAS model since we did not see interaction effect and model improvement in the VAS model. Models were adjusted for baseline demographics including age, sex, education, and BMI. Second, we used LMM with random intercept to identify factors associated with the HR-QoL trajectory. Covariates were introduced one at a time and model selection was based on Akaike Information Criterion (AIC) improvement. The final models included baseline age, sex, education, BMI, diabetes, cardiovascular history, institutionalisation, depression, and IADL. Third, we introduced a three-way interaction of amyloid status to clinical stage and time since enrollment to assess the effect of amyloid status on EQ-5D utility trajectory. We did not subclassify dementia into mild, moderate, and severe stages in the three-way interaction model due to few observations (< 3%) of moderate and severe dementia. Missing values were handled by LMM as missing at random. No multicollinearity was detected using the variance inflation factor. Model estimates and standard error (SE) were reported, and the significance level was set at *p* < 0.05. All analysis was done in R version 4.2.1.

## Results

### Baseline characteristics of the study population

Table 1 presents the baseline characteristics of the study population. Sixty percent of the participants had MCI at baseline. The mean age of the study population at baseline was 71 years, 62% were female, and nearly all patients were recruited from the community, i.e. no institutionalisation. As compared to patients with SCD, MCI patients have lower proportion of females (60%), fewer years of education (10.96 ± 3.13), lower MMSE scores (27.53 ± 2.12), higher depression scores (1.78 ± 3.16), and IADL (1.08 ± 0.2). A higher proportion of MCI patients smoke (8%) and have a cardiovascular history (15%) and diabetes (10%). Baseline health utilities were 0.84 ± 0.16 and 0.81 ± 0.18, and VAS were 75.8 ± 14.82 and 70.26 ± 15.77 in SCD and MCI patients. The mean follow-up duration was 4.2 years in SCD and 3.95 years in MCI with 3% of SCD and 21% of MCI patients developing dementia during the follow-up period.

**Table 1:** Characteristics of the study population

Variable	Total	SCD	MCI	*P* value
*N*	2255	919 (40%)	1336 (60%)	
Baseline age, mean (SD)	71.00 (8.67)	71.24 (8.14)	70.84 (9.02)	
Female, *n* (%)	1392 (62%)	592 (64%)	800 (60%)	< 0.05^b^
BMI, mean (SD)	25.55 (4.32)	25.43 (4.26)	25.64 (4.36)	
Education (year), mean (SD)	11.30 (3.04)	11.79 (2.83)	10.96 (3.13)	< 0.001^a^
No institutionalisation, *n* (%)	2250 (99%)	918 (99%)	1332 (99%)	
MMSE, mean (SD)	27.95 (1.90)	28.54 (1.32)	27.53 (2.12)	< 0.001^a^
Depression score, mean (SD)	1.52 (3.04)	1.14 (2.81)	1.78 (3.16)	< 0.001^a^
IADL, mean (SD)	1.06 (0.17)	1.03 (0.12)	1.08 (0.2)	< 0.001^a^
Smoking,* n* (%)	162 (7%)	49 (5%)	113 (8%)	< 0.05^b^
Alcohol units, mean (SD)	5.22 (7.86)	5.33 (7.30)	5.14 (8.23)	
Dyslipidemia, *n* (%)	629 (28%)	245 (27%)	384 (29%)	
Cardiovascular history, *n* (%)	307 (14%)	104 (11%)	203 (15%)	< 0.05^b^
Hypertension, *n* (%)	1362 (60%)	553 (60%)	809 (61%)	
Diabetes, *n* (%)	197 (9%)	66 (7%)	131 (10%)	< 0.05^b^
EQ-5D utility, mean (SD)	0.82 (0.17)	0.84 (0.16)	0.81 (0.18)	< 0.001^a^
EQ-5D VAS, mean (SD)	72.52 (15.63)	75.80 (14.82)	70.26 (15.77)	< 0.001^a^
Follow-up (year), mean (SD)	4.05 (1.64)	4.20 (1.58)	3.95 (1.67)	< 0.001^a^
Converts to dementia, n (%)	310 (14%)	31 (3%)	279 (21%)	< 0.001^b^

*SCD* Subjective cognitive decline, *MCI* Mild cognitive impairment, *SD* Standard deviation, *BMI* Body mass index, *MMSE* Mini-mental state examination, Depression, measured by neuropsychiatric inventory clinician-rated version (ranges from 0 to 21, higher scores indicate more depressive symptoms), *IADL* Instrumental activity of daily living (ranges from 1 to 4, higher scores indicate more functional impairment)

^a^Independent *t*-test

^b^Chi-square test

We also estimated the baseline EQ-5D utility and VAS for SCD and MCI patients with known amyloid status. We used the first available PET or CSF Aβ42 result to denote the amyloid status in 447 SCD and 454 MCI patients. The characteristics of the population are depicted in Table S1. There was no baseline difference in health utility between amyloid-negative and positive SCD and MCI patients (Table 2 and Fig. S1). The mean health utilities were 0.85 ± 0.14 and 0.86 ± 0.12 in amyloid-negative SCD and amyloid-positive SCD; 0.83 ± 0.17 and 0.84 ± 0.16 in amyloid-negative and amyloid-positive MCI. The VAS were 77.05 ± 14.34 and 75.75 ± 18.03 in amyloid-negative SCD and amyloid-positive SCD. There was a statistically significant difference (*P* < 0.05) in VAS between amyloid-negative (70.32 ± 15.88) and amyloid-positive MCI (73.41 ± 14.62).

**Table 2:** Mean baseline EQ-5D utility and VAS score in study population with known amyloid status (*n* = 901)

Variable	Amyloid negative SCD	Amyloid positive SCD	*P* value	Amyloid negative MCI	Amyloid positive MCI	*P* value
*N*	376	71		315	139	
EQ-5D utility, mean (SD)	0.85 (0.14)	0.86 (0.12)	0.56	0.83 (0.17)	0.84 (0.16)	0.59
EQ-5D VAS, mean (SD)	77.05 (14.34)	75.75 (18.03)	0.57	70.32 (15.88)	73.41 (14.62)	< 0.05

*SCD* Subjective cognitive decline, *MCI* Mild cognitive impairment, *SD* Standard deviation, *VAS* Visual analogue scale

### EQ-5D domain response

**Fig. 1:**
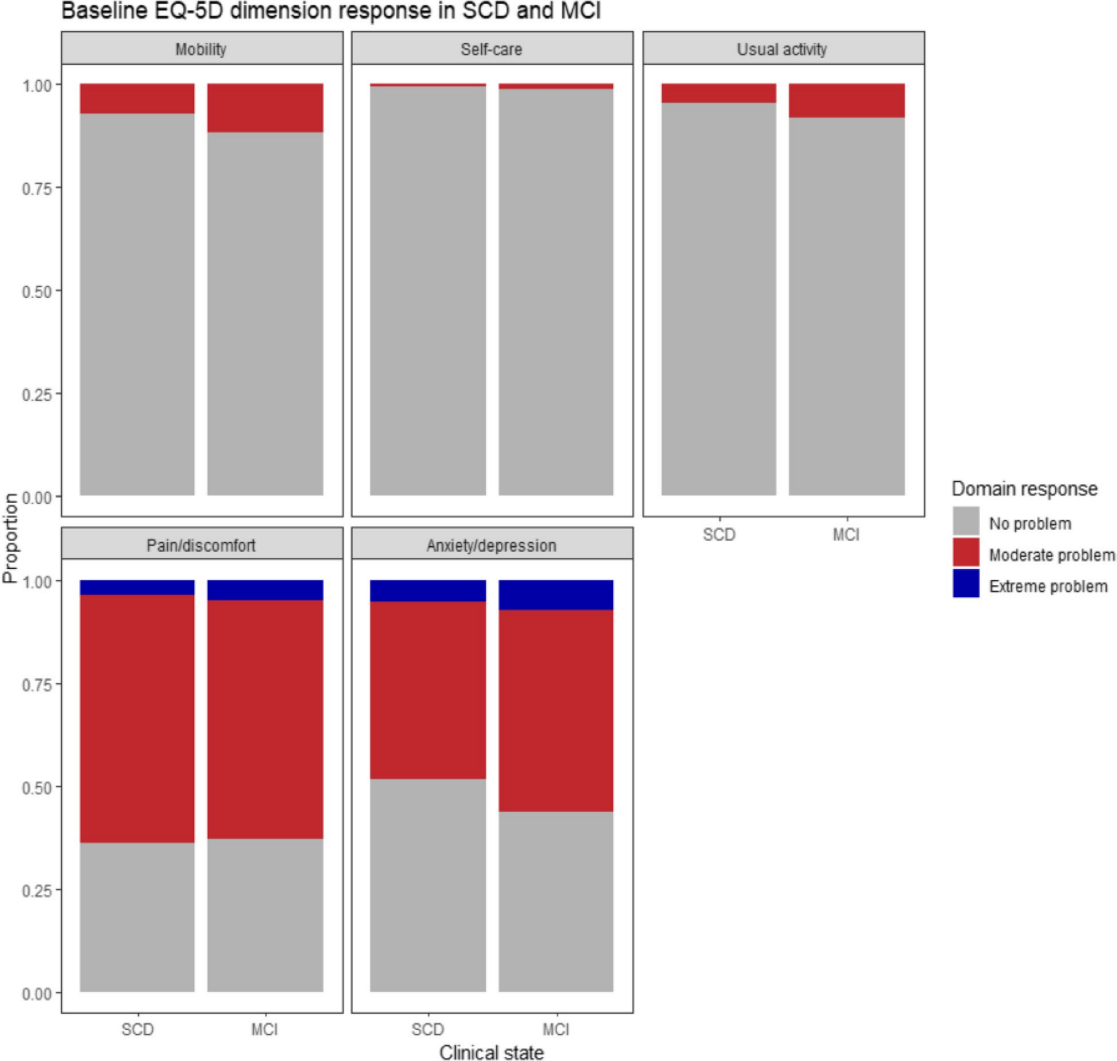
Baseline EQ-5D domain response in individuals with SCD and MCI

The responses to each EQ-5D domain by SCD and MCI at baseline were depicted in Fig. 1. A significantly higher proportion of MCI patients reported having some problems in walking about, performing usual activities, and having moderate to extreme anxiety/depression (*P* < 0.001) (Table S2). The self-care domain was relatively unaffected in both SCD and MCI patients. More than 60% of SCD and MCI patients reported having moderate to extreme pain and discomfort.

### HR-QoL trajectories

**Fig. 2:**
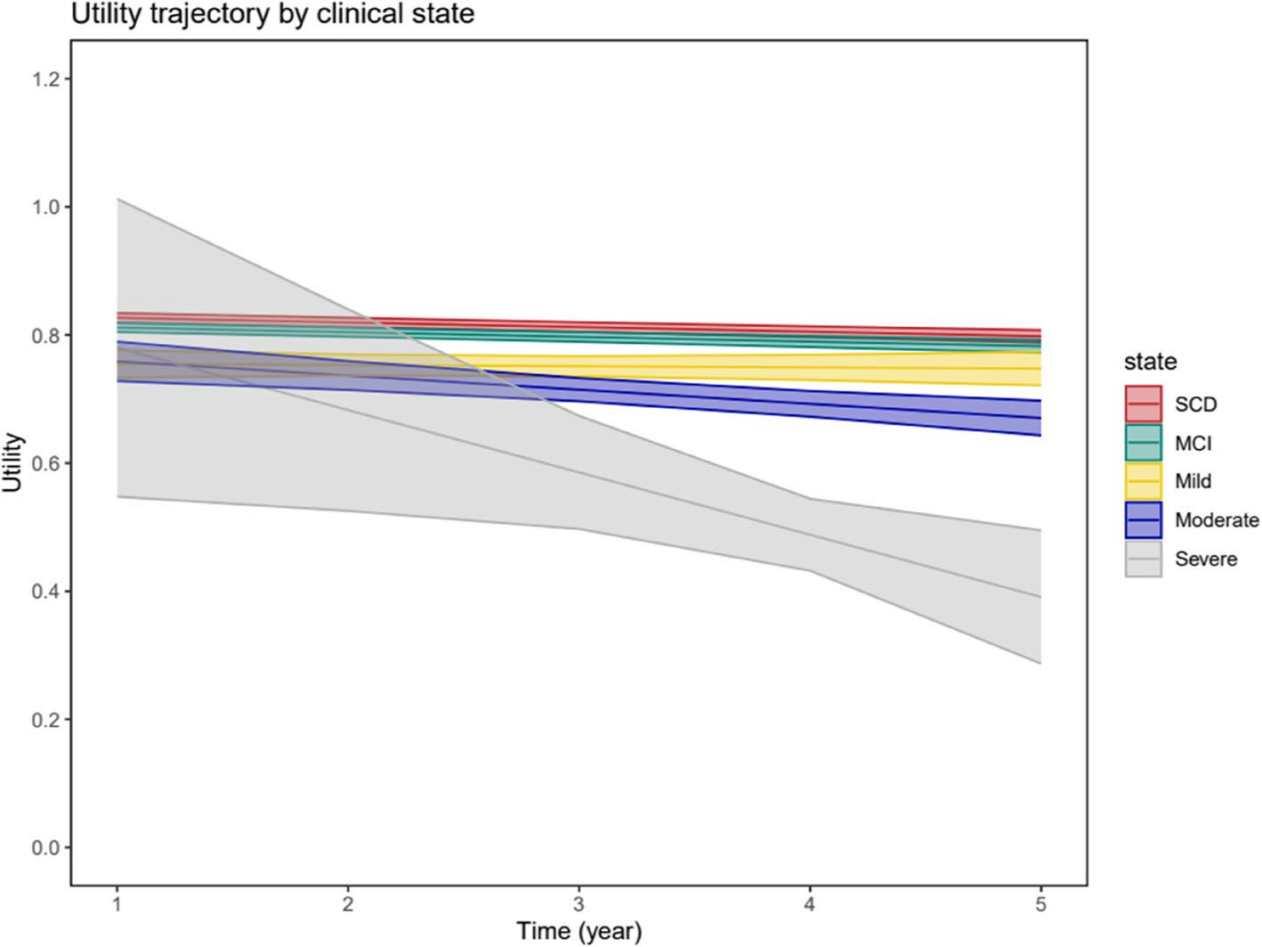
EQ5D-utility trajectory in subjective cognitive decline (SCD), mild cognitive impairment (MCI), mild, moderate, and severe dementia; linear-mixed effect model adjusted for baseline age, sex, education, and BMI

Table 3 and Fig. 2 show EQ-5D utility and VAS trajectory along the disease continuum after adjusting for baseline age, sex, education, and BMI. The clinical stage showed a significant time interaction effect on EQ-5D utility trajectory (*P* < 0.05). The annual change in health utility was − 0.015 (SE = 0.006) and − 0.09 (SE = 0.04) in moderate and severe dementia as compared to SCD. In the VAS model, the clinical stage showed a significant negative association with VAS (*P* < 0.001). The effect estimates were − 1.695 (SE = 0.274), − 4.401 (SE = 0.676), − 4.999 (SE = 0.8), and − 15.386 (SE = 3.142) in MCI, mild, moderate, and severe dementia. The annual decline in VAS was − 0.518 (SE = 0.062, *P* < 0.001).

**Table 3:** Linear mixed effect models showing EQ-5D utility and VAS trajectories

Variable	EQ-5D utility		VAS	
	Estimates (SE)	*P* value	Estimates (SE)	*P* value
Clinical stage		< 0.001		< 0.001
SCD	Reference		Reference	
MCI	− 0.015 (0.004)^a^		− 1.695 (0.274)^a^	
Mild	− 0.077 (0.015)^a^		− 4.401 (0.676)^a^	
Moderate	− 0.053 (0.021)^a^		− 4.999 (0.8)^a^	
Severe	0.043 (0.158)		− 15.386 (3.142)^a^	
Time (year)	− 0.007 (0.001)	< 0.001	− 0.518 (0.062)	< 0.001
Clinical stage × time interaction		< 0.05		
SCD × time	Reference			
MCI × time	0 (0.002)			
Mild × time	0.005 (0.005)			
Moderate × time	− 0.015 (0.006)^a^			
Severe × time	− 0.09 (0.04)^a^			

Models adjusted for baseline age, sex, education, and BMI. We did not include clinical stage and time interaction term in the VAS model since there was no interaction effect and no model improvement

*VAS* Visual analogue scale, *SCD* Subjective cognitive decline, *MCI* Mild cognitive impairment

^a^Statistically significant

### Factors associated with HR-QoL

The predictors for EQ-5D utility and VAS are depicted in Table 4. Older age was associated with lower health utility (estimates =  − 0.001, SE = 0.001, *P* < 0.05) but not VAS. Female sex and higher BMI were associated with lower health utility (estimate =  − 0.067, SE = 0.006, *P* < 0.001; estimate =  − 0.005, SE = 0.001, *P* < 0.001) and lower VAS (estimate =  − 1.743, SE = 0.562, *P* < 0.05; estimate =  − 0.122, SE = 0.063, *P* = 0.05). Diabetes is negatively associated with health utility (estimate =  − 0.032, SE = 0.011, *P* < 0.05) and VAS (estimate =  − 3.076, SE = 0.972, *P* < 0.05). Similarly, the presence of cardiovascular disease was negatively associated with health utility (estimate =  − 0.04, SE = 0.009, *P* < 0.001) and VAS (estimate =  − 2.784, SE = 0.791, *P* < 0.001). Institutionalisation was associated with lower health utility (estimate =  − 0.05, SE = 0.017, *P* < 0.05) but not with VAS. A higher depression score was associated with lower health utility (estimate =  − 0.014, SE = 0.001, *P* < 0.001) and VAS (estimate =  − 1.063, SE = 0.089, *P* < 0.001). Additionally, IADL was associated with a decline in health utility (estimates =  − 0.125, SE = 0.005, *P* < 0.001) and VAS (estimates =  − 3.009, SE = 0.459, *P* < 0.001) over time.

**Table 4:** Linear mixed effect models to identify predictors of HR-QoL

Variable	EQ-5D utility		VAS	
	Estimates (SE)	*P* value	Estimates (SE)	*P* value
Clinical stage		< 0.05		< 0.001
SCD	Reference		Reference	
MCI	− 0.012 (0.004)^a^		− 1.554 (0.286)^a^	
Mild	− 0.017 (0.015)		− 2.063 (0.777)^a^	
Moderate	0.03 (0.021)		− 1.308 (0.995)	
Severe	0.202 (0.155)		− 8.674 (3.27)^a^	
Time (year)	− 0.006 (0.001)	< 0.001	− 0.504 (0.065)	< 0.001
Clinical stage × time interaction		< 0.001		
SCD × time	Reference			
MCI × time	0.001 (0.002)			
Mild × time	0.02 (0.005)^a^			
Moderate × time	0.009 (0.006)			
Severe × time	− 0.057 (0.039)			
Baseline age	− 0.001 (0.001)	< 0.05	− 0.016 (0.032)	0.061
Sex female	− 0.067 (0.006)	< 0.001	− 1.743 (0.562)	< 0.05
Education (year)	0.001 (0.001)	0.23	0.172 (0.09)	0.06
BMI	− 0.005 (0.001)	< 0.001	− 0.122 (0.063)	0.05
Diabetes	− 0.032 (0.011)	< 0.05	− 3.076 (0.972)	< 0.05
Cardiovascular history	− 0.04 (0.009)	< 0.001	− 2.784 (0.791)	< 0.001
Institutionalisation	− 0.05 (0.017)	< 0.05	1.633 (1.542)	0.29
Depression	− 0.014 (0.001)	< 0.001	− 1.063 (0.089)	< 0.001
IADL	− 0.125 (0.005)	< 0.001	− 3.009 (0.459)	< 0.001

Models included diabetes, cardiovascular history, institutionalisation, depression, and IADL in addition to the baseline demographics. The purpose of the models was to identify predictors for HR-QoL. We did not interpret the estimates for the clinical stage since we included institutionalisation, depression, and IADL which are the consequences of cognitive decline (clinical stage in the model)

*SCD* Subjective cognitive decline, *MCI* Mild cognitive impairment, *BMI* Body mass index; Depression, measured by neuropsychological inventory clinician-rated version (higher scores indicate more symptoms), *IADL* Instrumental activities of daily living (higher scores indicate more functional impairment)

^a^Statistically significant

### Effect of amyloid status on health utility trajectory

**Fig. 3:**
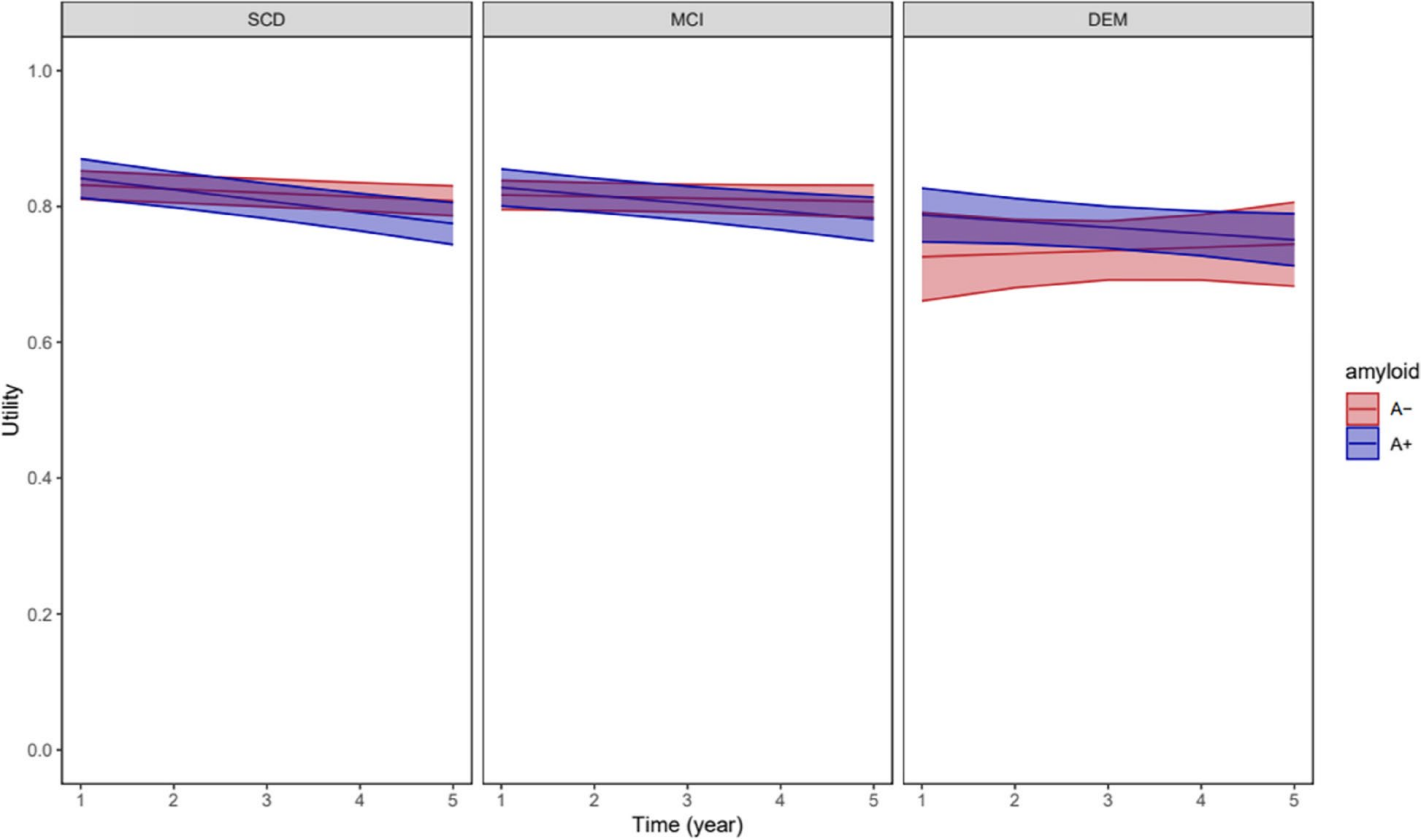
EQ-5D utility trajectory in amyloid-negative (A −) and amyloid-positive (A +) patients, linear-mixed effect model with three-way interaction of clinical stage × time × amyloid status, model adjusted for baseline age, sex, education, and BMI

Table 5 and Fig. 3 depict the LMM assessing the effect of amyloid status on EQ-5D utility trajectory. The model was adjusted for baseline age, sex, education, and BMI. There was no significant interaction between clinical stage and time, clinical stage and amyloid status, and the three-way interactions of clinical stage, time, and amyloid status. However, the interaction between time and amyloid status was significant (*P* < 0.05) indicating that amyloid-positive patients had a decline in health utility of − 0.011 (SE = 0.004) per year compared to amyloid-negative patients, irrespective of the clinical stage.

**Table 5:** Effect estimates for the impact of amyloid status on EQ-5D utility trajectory

Variable	Estimates (SE)	*P* value
Clinical stage × time		0.27
SCD × time	Reference	
MCI × time	0.003 (0.003)	
Dementia × time	0.007 (0.012)	
Clinical stage × amyloid		0.79
SCD × amyloid positive	Reference	
MCI × amyloid positive	0 (0.021)	
DEM × amyloid positive	0.031 (0.048)	
Time × amyloid positive	− 0.011 (0.004)	< 0.05
Clinical stage × time × amyloid		0.95
SCD × time × amyloid positive	Reference	
MCI × time × amyloid positive	0.002 (0.006)	
Dementia × time × amyloid positive	0.003 (0.014)	

Model adjusted for baseline age, sex, education, and BMI

*SCD* Subjective cognitive decline, *MCI* Mild cognitive impairment

## Discussion

### Discussion of findings

The study provides health utility estimates for a large cohort of SCD and MCI patients. The longitudinal study shows a significant decline in EQ-5D utility over time in moderate and severe dementia and a graded decrease in VAS with advancing clinical stages. Additionally, amyloid-positive patients have a steeper decline in health utility than amyloid-negative patients irrespective of the clinical stage. Older age, female sex, higher BMI, presence of diabetes, cardiovascular history, institutionalisation, higher depression score, and functional impairment are associated with poor HR-QoL.

We compare our estimated health utility for individuals with SCD to findings from two population-based surveys that utilised self-reported EQ-5D measures to study HR-QoL in the general population with SCD. The first study, conducted in Germany with a sample size of 3708, reported a health utility estimate of 0.89 (SD = 0.14) [32] while the second study, a nationwide cohort in Korea with a sample size of 37,364, reported an estimate of 0.83 (SD = 0.16) [33]. The main difference between our study and the aforementioned studies lies in the study design and sample population. Our study utilised a clinic-based cohort, where patients with cognitive complaints presented to memory clinics, while the two population-based surveys included more heterogeneous populations recruited in the community. Therefore, our estimated health utility of 0.84 is likely more representative of SCD individuals within the AD continuum.

The observed health utility in MCI patients can be compared to two studies that utilised patient-reported EQ-5D measures and shared the same study setting with ours [20, 34]. Heßmann et al. estimated a health utility of 0.72 (SD = 0.28) in 50 patients with a mean age of 76 years [20], while Jönsson et al. found an estimate of 0.84 in 47 patients with a mean age of 74 years [34]. The relatively lower health utility estimate in Heßmann et al.’s study might be explained by the older age and longer time (3.8 ± 4.4 years) spent in the disease stage. A systematic review indicates a wide variation of health utility estimates ranging from 0.72 to 0.89 in MCI patients depending on the study setting and population demographics [17]. Therefore, our estimate of 0.81 in the MCI population seems consistent with previous studies.

The health utilities from biomarker-informed SCD and MCI populations can be compared to the Swedish BioFINDER study, which shares a similar study setting and patient demographics to our study [18]. Our health utility estimates of 0.85 in amyloid-negative SCD and 0.86 in amyloid-positive SCD are comparable to the CU population in the Swedish BioFINDER study. However, the health utility estimates of 0.83 in amyloid-negative MCI and 0.84 in amyloid-positive MCI are higher than the corresponding estimates of 0.71 and 0.8 in the Swedish BioFINDER study. The differences between the two studies might be due to the differences in comorbidities and NPS shared by the two cohorts. Additionally, HR-QoL is a complex construct that can vary widely based on patient characteristics and other unmeasured factors, such as relationships with caregivers [35]. Therefore, the observed variation in health utility estimates between the two populations seems to be reasonable.

Domain analysis in our study shows that anxiety/depression and pain/discomfort are the most affected domains in individuals with SCD and MCI. The findings are consistent with the results from the BioFINDER study where more than 50% of participants reported having moderate to severe problems in the two domains [18]. Pain is a common non-specific symptom in the elderly population and is more commonly reported in SCD and MCI than in dementia [36]. The study also found that mobility, self-care, and usual activity domains were relatively unaffected, which is within expectation because patients with SCD and MCI can perform activities of daily living independently. These domains are typically affected in later disease stages when functional impairment becomes prominent. These findings provide valuable insights for understanding the impact of cognitive decline on HR-QoL domains in SCD and MCI stages.

The study reveals a faster decline of health utility in individuals with moderate and severe dementia over time and a constant decline in VAS with advancing clinical stages. The findings are consistent with previous research indicating that HR-QoL is lower with cognitive decline in individuals with SCD, MCI, and dementia [18, 20, 21]. However, the health utility decline in moderate and severe dementia was not significant anymore after adjusting for IADL (Table 4). In AD, cognitive decline precedes and predicts functional impairment [37] which manifests as limitations in complex IADLs such as medication intake, telephone use, and financial organisation in the early stage of the disease [38]. As the disease progresses, functional limitation is prominent in basic functions such as eating, dressing, and toileting leading to care dependency and poor HR-QoL [38]. A study by Janssen et al. on people at risk of dementia shows that the association between cognition and HR-QoL is mediated by IADL [39]. The steeper decline of health utility in moderate and severe dementia might be explained by the mediation effect of prominent functional impairment in these stages.

Similar to IADL, Janssen et al. have shown that depression is a mediator between the association of cognition and HR-QoL [39]. Our study shows a negative association between depressive symptoms and HR-QoL, which concurs with findings from previous research in SCD and MCI populations [18, 33]. Depression is a core NPS in AD and related dementias [40]. It typically manifests early in cognitive decline and is linked to disease progression and functional impairment [41]. Moreover, depression usually coexists with SCD, and both are early manifestations of AD and related dementia [42]. The study highlights the importance of considering depression in studying HR-QoL in AD.

The results of our study confirm previous findings that older age, female sex [32, 43, 44], institutionalisation, and functional dependence [20, 21] are predictors for lower HR-QoL. The higher depression scores in our study might explain the lower HR-QoL in females (Table S3 in additional file). This finding aligns with previous research indicating that females tend to report higher SCD and depressive symptoms [1] and patient-rated depressive symptoms are an independent predictor of HR-QoL [3]. We did not see any association between education and HR-QoL. The association between education and HR-QoL are conflicting in previous studies with a cross-sectional study showing a positive association [32] while a longitudinal study showed no association in SCD populations [43]. The association in our study might have been attenuated by the influence of other key factors of dementia like depression and IADL. We also found that modifiable risk factors for dementia development such as diabetes, cardiovascular diseases, and obesity were associated with poor HR-QoL in SCD and MCI patients. This suggests that multidomain health interventions targeting these modifiable risk factors to prevent dementia development [45] may also help to maintain HR-QoL in individuals with SCD.

Furthermore, our study reveals a faster decline of HR-QoL in amyloid-positive than amyloid-negative patients. This could be explained by a faster decline in cognitive and functional ability in individuals with amyloid-positive findings. A recent longitudinal study using data from Amsterdam Dementia Cohort also showed a faster decline of HR-QoL in amyloid-positive SCD and MCI patients [19], although a direct comparison between the two studies is challenging due to differences in the analysis approach. Further longitudinal studies investigating the relationship between amyloid status and HR-QoL are needed.

### Strengths and limitations

The study population comes from the nationwide memory clinic cohort with specific diagnostic criteria for SCD and MCI populations. The utility estimated from this cohort is representative of the SCD and MCI population and can be applied directly to the health economic models evaluating interventions in these populations. Moreover, we present utility for both general estimates and biomarker-confirmed SCD and MCI patients, allowing researchers to choose from the different estimates based on the target populations. In addition, the study’s longitudinal design provides insight into the HR-QoL trajectory along the disease continuum and allows us to establish the relationship between predictors for HR-QoL in the early stage of AD.

The limitation of this study is the use of patient-rated EQ-5D. Although patients with SCD and MCI can be considered competent to rate their own HR-QoL, studies suggested that patient-rated HR-QoL is consistently higher than the proxy-rated version in the dementia stage [17]. Using patient-rated EQ-5D to assess the HR-QoL as the disease progresses might underestimate the magnitude of HR-QoL decline over time. Another limitation relates to the optional nature of biomarker testing within our cohort. Notably, individuals who underwent biomarker testing in our study displayed a healthier profile compared to those who did not receive this testing (Table S4 of additional file). Consequently, there is a possibility that HR-QoL estimates for this subgroup may overstate the HR-QoL within the broader population. However, it is worth noting that we also provided HR-QoL estimates for the entire cohort, aiming to ensure that our findings are representative of the SCD and MCI populations, whether or not they underwent biomarker testing. Additionally, longitudinal studies involving dementia patients often encounter issues associated with selective dropout where patients with severe disease are more likely to be lost to follow-up which might further lead to an underestimation of HR-QoL decline in the severe disease stage.

## Conclusion

The study provides health utility estimates for SCD and MCI which can be applied in the economic evaluations of interventions targeting these populations. Health utility declines over time in moderate and severe dementia, and VAS declines with advancing clinical stages. Amyloid-positive patients experience a faster decline in health utility than amyloid-negative patients, indicating the importance of considering biomarker status in HR-QoL assessments. Future research is needed to confirm the longitudinal relationship between amyloid status and HR-QoL and to examine the level at which depression and IADL contribute to the HR-QoL decline in AD and related dementias.

### Supplementary Information

**Additional file 1:** **Table S1.** Characteristics of the study population with known amyloid status (*n *= 901). **Figure S1.** Mean baseline EQ-5D utility and VAS score in the study population with known amyloid status. **Table S2.** Baseline EQ-5D domain responses. **Table S3.** Sex differences on the characteristics of the study population. **Table S4.** Differences in clinical characteristics based on receiving biomarker testing or not.

## Data Availability

The datasets generated during and/or analysed in the current study are not publicly available due to the involvement of patient information but are available from the corresponding author upon reasonable request.
