# Supplementary material for: Health-related quality of life in subjective cognitive decline and mild cognitive impairment: a longitudinal cohort analysis
Source: Alzheimers Res Ther. 2023 Nov 15;15:200. doi: 10.1186/s13195-023-01344-0 (PMC10648708; doi:10.1186/s13195-023-01344-0)
Supplement: Supplementary file 1 — Additional file 1: Table S1. Characteristics of the study population with known amyloid status (n = 901). Figure S1. Mean baseline EQ-5D utility and VAS score in the study population with known amyloid status. Table S2. Baseline EQ-5D domain responses. Table S3. Sex differences on the characteristics of the study population. Table S4. Differences in clinical characteristics based on receiving biomarker testing or not. [file 13195_2023_1344_MOESM1_ESM.docx]

**Supplementary material: This file contains additional information to the manuscript** “Health-related quality of life in subjective cognitive decline and mild cognitive impairment: a longitudinal cohort analysis”

Table S1. Characteristics of the study population with known amyloid status (n=901)

| Variable | Amyloid negative SCD | Amyloid positive SCD | Amyloid negative MCI | Amyloid positive MCI |
| --- | --- | --- | --- | --- |
| N | 376 | 71 | 315 | 139 |
| Baseline age, mean (SD) | 71.44 (8.13) | 75.49 (6.14) | 67.61 (8.49) | 72.38 (6.85) |
| Female, n (%) | 238 (63%) | 41 (58%) | 176 (56%) | 66 (47%) |
| BMI, mean (SD) | 25.58 (3.87) | 25.04 (3.50) | 25.56 (4.33) | 24.96 (3.59) |
| Education (year), mean (SD) | 12.09 (2.70) | 12.01 (2.74) | 11.17 (3.04) | 10.99 (3.17) |
| No institutionalization, n (%) | 376 (100%) | 70 (99%) | 314 (100%) | 139 (100%) |
| MMSE, mean (SD) | 28.64 (1.09) | 28.50 (1.15) | 27.94 (1.84) | 26.82 (2.34) |
| Depression, mean (SD) | 0.89 (2.52) | 0.54 (1.34) | 1.71 (2.85) | 1.40 (2.88) |
| IADL, mean (SD) | 1.04 (0.12) | 1.05 (0.15) | 1.04 (0.13) | 1.06 (0.13) |
| Dyslipidemia, n (%) | 99 (26%) | 16 (23%) | 90 (29%) | 42 (30%) |
| Cardiovascular history, n (%) | 37 (10%) | 11 (15%) | 42 (13%) | 22 (16%) |
| Hypertension, n (%) | 240 (64%) | 47 (66%) | 185 (59%) | 81 (58%) |
| Diabetes, n (%) | 22 (6%) | 6 (8%) | 29 (9%) | 11 (8%) |
| Smoking, n (%) | 22 (6%) | 6 (8%) | 31 (10%) | 10 (7%) |
| Alcohol units, mean (SD) | 6.17 (7.58) | 5.37 (8.03) | 5.53 (8.63) | 3.96 (5.17) |
| Follow up (year), mean (SD) | 4.49 (1.30) | 4.35 (1.34) | 4.61 (1.09) | 4.34 (1.34) |
| Converts to dementia, n (%) | 4 (1%) | 9 (13%) | 31 (10%) | 76 (55%) |
| Note. SCD – subjective cognitive decline, MCI – mild cognitive impairment, SD – standard deviation, BMI – body mass index, MMSE – mini-mental state examination, Depression - measured by neuropsychological inventory clinician-rated version (ranges from 0 to 21, higher scores indicate more symptoms), IADL – instrumental activity of daily living (ranges from 1 to 4, higher scores indicate more functional impairment). | | | | |

Figure S1. Mean baseline EQ-5D utility and VAS score in the study population with known amyloid status


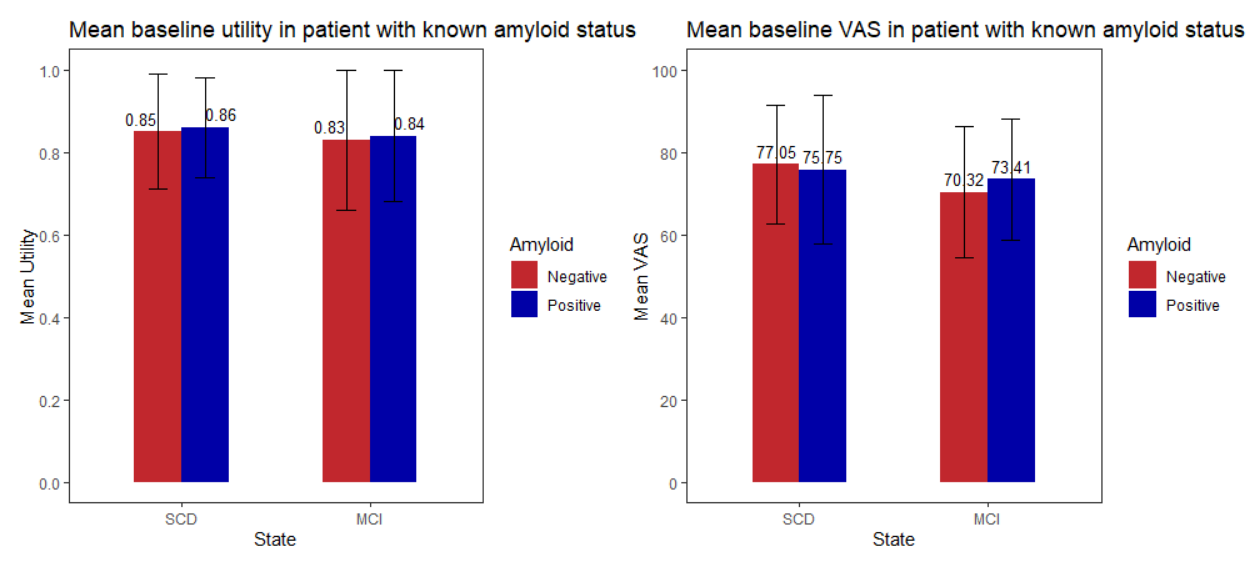


Table S2. Baseline EQ-5D domain responses

| EQ5D domain | Response | SCD (%) | MCI (%) | P value |
| --- | --- | --- | --- | --- |
| Mobility | No problem in walking about | 852 (93%) | 1176 (88%) | <0.001 |
|  | Some problems in walking about | 67 (7%) | 159 (12%) |  |
|  | Confined to bed | 0 (0%) | 0 (0%) |  |
| Self-care | No problem with self-care | 914 (99%) | 1320 (99%) | 0.149 |
|  | Some problems in washing or dressing myself | 5 (1%) | 15 (1%) |  |
|  | Unable to wash or dress myself | 0 (0%) | 0 (0%) |  |
| Usual activity | No problem with performing usual activities | 878 (96%) | 1224 (92%) | <0.001 |
|  | Some problems in performing usual activities | 41 (4%) | 110 (8%) |  |
|  | Unable to perform usual activities | 0 (0%) | 0 (0%) |  |
| Pain/ discomfort | No pain or discomfort | 332 (36%) | 493 (37%) | 0.258 |
|  | Moderate pain or discomfort | 552 (60%) | 773 (58%) |  |
|  | Extreme pain or discomfort | 35 (4%) | 69 (5%) |  |
| Anxiety/ depression | Not anxious or depressed | 474 (52%) | 581 (44%) | <0.001 |
|  | Moderately anxious or depressed | 396 (43%) | 654 (49%) |  |
|  | Extremely anxious or depressed | 49 (5%) | 99 (7%) |  |

Table S3. Sex differences on the characteristics of the study population

| Variable | Total | Female | Male | P value |
| --- | --- | --- | --- | --- |
| N | 2255 | 1392 (62%) | 863 (38%) |  |
| Baseline age, mean (SD) | 71.00 (8.67) | 70.73 (8.85) | 71.44 (8.36) |  |
| BMI, mean (SD) | 25.55 (4.32) | 25.20 (4.65) | 26.12 (3.66) | <0.001† |
| Education (year), mean (SD) | 11.30 (3.04) | 11.08 (3.11) | 11.66 (2.90) | <0.001† |
| No Institutionalization, n (%) | 2250 (99.9) | 1388 (99.9) | 862 (99.9) |  |
| MMSE, mean (SD) | 27.95 (1.90) | 27.96 (1.93) | 27.92 (1.84) |  |
| Depression score, mean (SD) | 1.52 (3.04) | 1.68 (3.20) | 1.26 (2.73) | <0.001† |
| IADL, mean (SD) | 1.06 (0.17) | 1.04 (0.13) | 1.09 (0.22) | <0.001† |
| Smoking, n (%) | 162 (7) | 95 (7) | 67 (8) |  |
| Alcohol units, mean (SD) | 5.22 (7.86) | 3.26 (5.18) | 8.39 (10.12) | <0.001† |
| Dyslipidemia, n (%) | 629 (28) | 356 (26) | 273 (32) | <0.05* |
| Cardiovascular history, n (%) | 307 (14) | 136 (10) | 171 (20) | <0.001* |
| Hypertension, n (%) | 1362 (60) | 790 (57) | 572 (66) | <0.001* |
| Diabetes, n (%) | 197 (9) | 86 (6) | 111 (13) | <0.001* |
| EQ-5D utility, mean (SD) | 0.82 (0.17) | 0.80 (0.18) | 0.86 (0.15) | <0.001† |
| EQ-5D VAS, mean (SD) | 72.52 (15.63) | 71.74 (16.10) | 73.78 (14.75) | <0.001† |
| Follow-up (year), mean (SD) | 4.05 (1.64) | 4.01 (1.68) | 4.12 (1.57) |  |
| Converts to dementia, n (%) | 310 (14) | 165 (12) | 145 (17) | <0.05* |
| SCD (%) | 919 (41) | 592 (43) | 327 (38) | <0.05* |

Note. SCD – subjective cognitive decline, MCI – mild cognitive impairment, SD – standard deviation, BMI – body mass index, MMSE – mini-mental state examination, Depression – measured by neuropsychiatric inventory clinician-rated version (ranges from 0 to 21, higher scores indicate more depressive symptoms), IADL – instrumental activity of daily living (ranges from 1 to 4, higher scores indicate more functional impairment), † - independent t-test, * - chi-square test.

Table S4. Differences in clinical characteristics based on receiving biomarker testing or not

| Variable | Total | No biomarker | Biomarker | P value |
| --- | --- | --- | --- | --- |
| N | 2255 | 1354 (60%) | 901 (40%) |  |
| Baseline age, mean (SD) | 71.00 (8.67) | 71.29 (8.92) | 70.57 (8.28) | <0.05† |
| Female, n (%) | 1392 (62) | 871 (64) | 521 (58) | <0.05* |
| BMI, mean (SD) | 25.55 (4.32) | 25.63 (4.54) | 25.44 (3.97) |  |
| Education (year), mean (SD) | 11.30 (3.04) | 11.11 (3.09) | 11.59 (2.94) | <0.001† |
| No Institutionalization, n (%) | 2250 (99.99) | 1351 (99.99) | 899 (99.99) |  |
| MMSE, mean (SD) | 27.95 (1.90) | 27.84 (1.99) | 28.10 (1.73) | <0.001† |
| Depression score, mean (SD) | 1.52 (3.04) | 1.74 (3.27) | 1.22 (2.65) | <0.001† |
| IADL, mean (SD) | 1.06 (0.17) | 1.07 (0.20) | 1.04 (0.13) | <0.05† |
| Smoking, n (%) | 162 (7) | 93 (7) | 69 (8) |  |
| Alcohol units, mean (SD) | 5.22 (7.86) | 5.00 (7.96) | 5.54 (7.72) |  |
| Dyslipidemia, n (%) | 629 (28) | 382 (28) | 247 (27) |  |
| Cardiovascular history, n (%) | 307 (14) | 195 (14) | 112 (12) |  |
| Hypertension, n (%) | 1362 (60) | 809 (60) | 553 (61) |  |
| Diabetes, n (%) | 197 (9) | 129 (10) | 68 (8) |  |
| EQ-5D utility, mean (SD) | 0.82 (0.17) | 0.81 (0.19) | 0.84 (0.15) | <0.001† |
| EQ-5D VAS, mean (SD) | 72.52 (15.63) | 71.51 (15.63) | 74.04 (15.51) | <0.001† |
| Follow-up (year), mean (SD) | 4.05 (1.64) | 3.75 (1.80) | 4.50 (1.24) | <0.001† |
| Converts to dementia, n (%) | 310 (14) | 190 (14) | 120 (13) |  |
| SCD (%) | 919 (41) | 472 (35) | 447 (50) | <0.001* |

Note. SCD – subjective cognitive decline, SD – standard deviation, BMI – body mass index, MMSE – mini-mental state examination, Depression – measured by neuropsychiatric inventory clinician-rated version (ranges from 0 to 21, higher scores indicate more depressive symptoms), IADL – instrumental activity of daily living (ranges from 1 to 4, higher scores indicate more functional impairment), † - independent t-test, * - chi-square test.
